# Supplementary material for: Genomic insights into adaptive divergence and speciation among malaria vectors of the Anopheles nili group
Source: Evol Appl. 2017 Jun 12;10(9):897–906. doi: 10.1111/eva.12492 (PMC5680430; doi:10.1111/eva.12492)
Supplement: Supplementary file 1 [file EVA-10-897-s001.docx]

**Supplemental information**

**Table S1:** Information on mosquito samples included in this study.

|  |  |  |  |  |  |  |
| --- | --- | --- | --- | --- | --- | --- |
| **Sampling** | **Geographic** | **Sampling** | | | **Total** |  |
| **location** | **coordinates** | **method** | | |  |  |
|  |  | **HLC-OUT** | **HLC-IN** | **LC** |  |  |
| Ebebda | 4°20'00"N, 11°17'00"E |  |  | 6 | 6 |  |
| Nkoteng | 4°31'00"N, 12°02'00"E |  |  | 8 | 8 |  |
| Nyabessan | 2°24'00"N, 10°24'00"E | 63 | 44 |  | 107 |  |
| Mbébé | 4°10'00"N, 11°04'00"E | 13 | 3 | 8 | 24 |  |
| Total |  | 76 | 47 | 22 | 145 |  |
|  |  |  |  |  |  |  |
| HLC-OUT, human landing catches performed outdoor; HLC-IN, human landing catches performed indoor; LC, larval collection | | | | | | |
